# Analysis of Cochlear Parameters in Paediatric Inner Ears with Enlarged Vestibular Aqueduct and Patent Cochlea

**DOI:** 10.3390/jpm12101666

**Published:** 2022-10-07

**Authors:** Jianan Li, Shuoshuo Kang, Haiqiao Du, Shuwei Wang, Dandan Wang, Mengyu Liu, Shiming Yang

**Affiliations:** 1 Senior Department of Otolaryngology-Head & Neck Surgery, The Sixth Medical Center of Chinese PLA General Hospital, Beijing 100853, China; 2 National Clinical Research Center for Otolaryngologic Diseases, Beijing 100853, China; 3 State Key Lab of Hearing Science, Ministry of Education, Beijing 100853, China; 4 Department of Radiology, The First Medical Center of Chinese PLA General Hospital, Beijing 100853, China

**Keywords:** high-resolution computed tomography, enlarged vestibular aqueduct, inner ear, cochlear implant

## Abstract

Is cochlear implant (CI) electrode selection for cochleae with an enlarged vestibular aqueduct (EVA) the same as that for patent cochleae with a normal inner ear structure? Preoperative high-resolution computed tomography (HRCT) images of 247 ears were assessed retrospectively. The A-value, B-value, and H-value were measured with OTOPLAN, and Bell curves were created to show the distribution. All ears with EVA were re-evaluated using a 3D slicer to confirm whether incomplete partition type II (IP II) existed. The Mann–Whitney U-test was applied to determine a statistically significant difference. After adjustment with the Bonferroni correction method, a *p*-value ≤ 0.006 was considered significant. In total, 157 ears with patent cochlea and 90 ears with EVA were assessed. Seventy (82%) of the EVA ears had an IP II malformation, and 14 (19%) of these were not detected by CT scan but were later seen through the 3D reconstruction. A significant difference was found for the A value and B value between the patent cochleae and EVA-only and between the patent cochleae and EVA with IP II. Most EVA cases had an IP II malformation. The basal turn of the cochlea may be smaller in EVA cases than in the patent cochleae. Electrode selection should be adjusted accordingly.

## 1. Introduction

An enlarged vestibular aqueduct (EVA) is a congenital malformation that is most frequently diagnosed in children with hearing loss [1]. An EVA often entails additional anatomical abnormities, such as an enlarged vestibule, enlarged semi-circular canal, incomplete partition type II (IP II), or cochlear hypoplasia [2].

The visualisation of an inner ear malformation from radiographs is a challenging procedure [3]. To mitigate this, 3D segmentation has established itself in clinical practice as a routine imaging technique for the visualization of anatomical inner ear structures after the CT imaging has shown abnormities [3]. The measurement of the cochlear diameter (A-value) from the pre-operative imaging is useful in the assessment of the cochlear size in those with a normal inner ear anatomy; however, it is not applicable in cases with inner ear malformation [4]. The electrode array length selection based on the A-value measurement, which is a part of the pre-operative image assessment, is only valid for cochleae with 2 ½ turns and not for cochleae with a cystic apex. It is essential to differentiate anatomically normal cochleae from cystic cochleae.

Electrode array length selection in cases with inner ear malformation should be conducted carefully to avoid selecting an electrode that is too long for the cystic apex. It has been reported that inserting a longer length electrode into cochleae with a cystic apex has resulted in electrode tip fold-over [5]. Only appropriate imaging and visualization of malformed cochleae facilitate the appropriate electrode array selection, which in turn enhances cochlear implant recipients’ quality of life (QoL).

The CI is the gold-standard treatment option for the sensorineural hearing loss condition. The incidence of inner ear malformation is at a rate of 20–30% among the population with congenital hearing loss and 30% in the Chinese population, according to a study on 2747 cases of inner ear malformation for the classification of patients with sensorineural hearing loss [6].

OTOPLAN^®^ is an otological planning software developed by the Cascination AG, which is CE-marked for use in clinical practice to assess the cochlear size from temporal bone images. The software requires the user to provide defined anatomical landmarks capturing the diameter (A-value) and width (B-value: cochlear width perpendicular to the line segment of the A-value intersecting at the mid-modiolus) of the cochlear basal turn in the oblique coronal view, which is obtained by rotating the axial, coronal, and sagittal axes until the basal turn is fully captured [7]. A third parameter (H-value) defines the height of the cochlea between the apex and the base of the cochlea through the modiolus perpendicular to A and B.

This study aimed to (a) assess the cochlear dimensions in Chinese paediatric CI candidates with fully developed patent cochleae and with EVA using the OTOPLAN software and to (b) analyse the differences between side of the ear (left/right), sex, and type of malformation.

## 2. Materials and Methods

### 2.1. Inclusion and Exclusion Criteria

To be eligible for enrolment in the study, the subjects were required to be aged 18 or younger, to be candidates for implantation with a MED-EL CI at the study site, and to be diagnosed with either a fully developed (patent) cochlea or EVA.

Subjects were not eligible for enrolment in the study if they had been diagnosed with any type of inner ear malformation other than EVA via CT or MRI.

### 2.2. Study Conduct

This observational retrospective cohort study was conducted between 2017 and 2020 at the Institute of Otolaryngology at the Chinese PLA General Hospital in Beijing, China.

**Figure 1:**
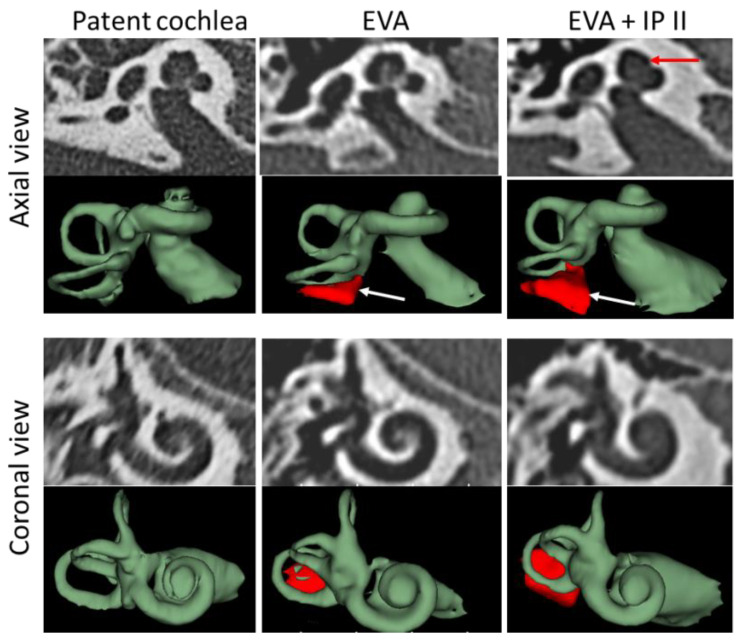
Visualisation of different cochlear anatomies. The red arrow in the EVA + IP II column points to the cystic apex. The white arrows in the second row point to the enlarged vestibular aqueducts.

The cochlear parameters in subjects with EVA and patent cochleae were analysed to determine whether EVA constitutes a challenge when selecting an appropriate electrode array. For this, high-resolution computed tomography (HRCT) scans of the temporal bone were reviewed and then imported to the OTOPLAN software. The A-value (diameter), B-value (width), and H-value (height) were measured using OTOPLAN (version 1.2). A 3D slicer (https://www.slicer.org/, accessed on 4 November 2021) was used to segment the complete inner ear to identify the EVA and cystic apex following the 3D reconstruction procedure described elsewhere (3). Figure 1 depicts the visualizations of the different cochlear anatomies using CT images and 3D images in both axial and coronal view.

Data were analysed as per side of the ear (left/right), sex, and type of malformation.

### 2.3. Statistical Analysis

The mean, the standard deviation (SD), and/or the median with range (minimum and maximum values) were used to describe the patient characteristics and characteristics the of the cochlea dimensions. For qualitative data, absolute and relative frequencies were used.

The Mann–Whitney U-test was applied to statistically evaluate the differences in the cochlea dimensions between the CI candidates with fully developed cochlea and those with EVA. The Kolmogorov–Smirnov and the Shapiro–Wilk tests were used to check the data distribution. Bell curves were created to show the distribution.

The statistical significance was set to *p*-values ≤ 0.05. To control for the problem of multiplicity resulting from multiple comparisons (i.e., to avoid the Type I error), *p*-values were adjusted by applying the Bonferroni correction method. Hence, a *p*-value ≤ 0.006 instead of ≤0.05 was considered as significance level.

IBM SPSS Statistics Version 25 (IBM, Armonk, NY, USA) was used for the analyses.

## 3. Results

### 3.1. Subject Demographics

In total, 124 subjects satisfied both the inclusion and exclusion criteria between April 2017 and May 2020. Of these, 116 had a FLEX 28 implanted. From those, 247 ears were analysed using OTOPLAN^®^. In total, 157 ears with patent cochleae and 90 ears with EVA were included in the data analysis. Out of the 90 EVA cases, 16 ears displayed isolated EVA (EVA only), and 74 ears had EVA combined with IP II (EVA + IP II). A total of 14 (19%) of the IP II cases were detected using the 3D reconstruction.

The mean age at surgery was 2.5 years (0.6–12.8 years) in the patent cochlea group, 3.1 years (0.0–10.8 years) in the EVA only group, and 4.7 years (0.0–18.3 years) in the EVA + IP II group.

### 3.2. Cochlear Parameters

For the comparison of the mean A, H, and B values between the three different groups, see Table 1.

**Figure 2:**
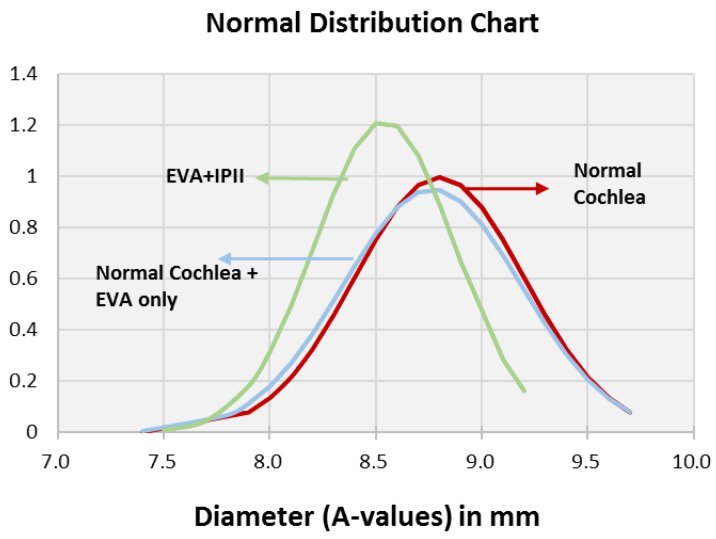
Bell curve (normal distribution curve) of the A value comparing the groups with normal cochleae, normal cochleae + EVA only, and EVA + IP II.

**Figure 3:**
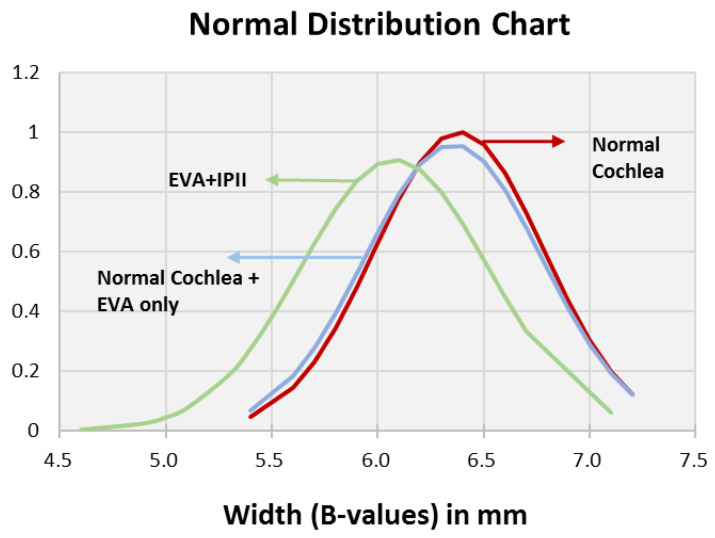
Bell curve (normal distribution curve) of the B value comparing the groups with normal cochleae, normal cochleae + EVA only, and EVA + IP II.

**Figure 4:**
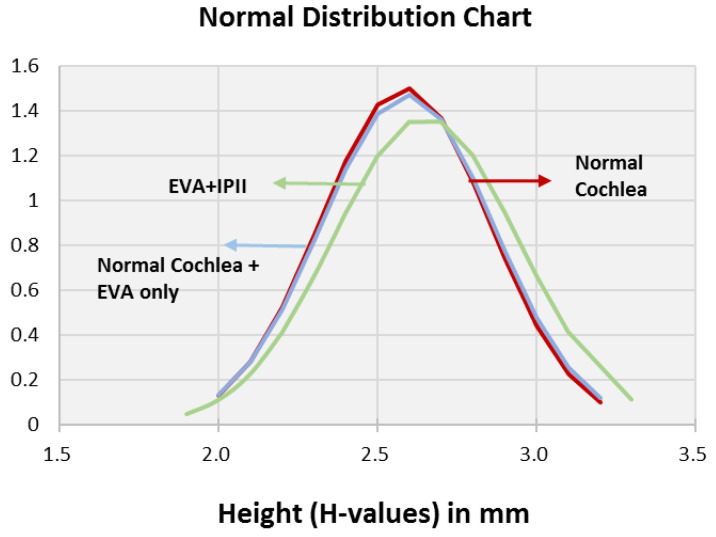
Bell curve (normal distribution curve) of the H value comparing the groups with normal cochleae, normal cochleae + EVA only, and EVA + IP II.

The distributions of the A and B values were similar for the normal cochleae and EVA only (Figure 2 and Figure 3), whereas the EVA + IP II significantly differed from them (*p* < 0.0001). The A values and B values did not significantly differ between the EVA only and the EVA + IP II group (*p* = 0.494) (Figure 2 and Figure 3). The H value did not differ significantly between any of the three groups (*p* = 0.259 for the comparison between patent cochleae and EVA only; *p* = 0.091 for patent cochleae and EVA + IP II; *p* = 0.738 for EVA only and EVA + IP II). (Figure 4).

In total, 37.6% (59/159) of the patent cochleae, 12.5% (2/16) of the EVA only group, and 9.46% (7/74) of the EVA + IP II group had an A value of ≥9.0 mm.

No significant differences were found between the left and right ears in the three groups of patent cochleae, EVA only, and EVA with IP II (Table 2).

No significant differences were found between males and females in any of the three groups (see Table 3).

## 4. Discussion

This study showed how much cochlear size varied among Chinese paediatric CI candidates and thus the requirement for electrodes arrays of different lengths in this population. The current study also clearly showed that most EVA cases (82%) were combined with IP II malformation, and the basal turn diameter of the cochlea may be smaller in EVA cases than in patent cochleae. This requires adjusted electrode array selection. IP II may not be easily diagnosed with plain CT views; therefore, 3D reconstruction may be helpful to ensure the correct diagnosis.

The fact that more than one third of the patent cochleae group had a basal turn diameter of 9 mm or longer showed that electrode arrays shorter than 30 mm would not cover the entire frequency range, and that long electrode arrays for complete cochlear coverage remain essential to ideally restore hearing in those profound deaf CI recipients [8]. The fact that 12.5% of the EVA only group and almost 10% of the EVA + IP II also had a cochlear basal turn diameter ≥9 mm means that care should be taken not to overrule the cystic apex while selecting the electrode array length. The cystic cochlear apex may not assist in the optimal placement of the longer length electrode in the second turn of the cochlea, as the risk of electrode tip fold-over is higher [5]. Higher mean A-values in the anatomically normal and isolated EVA cochleae than in subjects with IP type I, II, III or cochlear hypoplasia were also observed in a recent study by Khurayzi et al. [3].

The 3D segmented image of the inner ear shows the presence of EVA and cystic apex to an extent that could help the assessing clinician to conduct an in-depth image analysis both in terms of optimal electrode array selection and in the case of no prior detection of inner ear malformation.

Preoperatively, it is better to have a backup device with an electrode array variant that has a conical stopper, such as the commercially available FORM electrode arrays, especially for cases with EVA and EVA + IP II. The FORM electrode array could be chosen once the cochlea has been opened and seen with the cerebrospinal fluid (CSF) gushing or oozing. For the EVA only, the insertion of a standard electrode array is recommended if no gushing occurs after the cochlear opening. For EVA only, with gushing after the cochlea opening, a FORM electrode array of length 24 mm may be a solution based on our experience. For the EVA + IP type II, a FORM 24 array or a FORM 19 array (19 mm) may be a solution depending on the overall size of the cochlea. As per the report of Sennaroglu et al., the FORM electrode with cork type stopper provides necessary sealing to the cochlear opening thereby minimizing or stopping the CSF leakage [7].

In summary, EVA cases are often combined with IP II, which may be missed from a plain CT view but can be diagnosed with 3D reconstruction. OTOPLAN is a helpful tool to estimate the length of the electrode array for normal cochleae. In cases of EVA combined with IP II, a FORM electrode should be prepared as a backup in the operating room and could be used in case of CSF oozing after cochleostomy. The length of the electrode array could be shorter than the OTOPLAN recommendation to avoid tip fold-over.

Future studies are needed to investigate tip fold-over in EVA + IP II patients implanted with long electrode arrays. Electrode tip fold-over is related more to pre-curved electrodes and is rather rare with straight lateral wall electrodes in patent cochleae [8]. Further investigation is still needed to identify the frequency of tip fold over in IP II cases with FLEX electrodes. The current study shows that the FLEX 28 can be a good solution for IP II cases if it is carefully inserted. The FORM electrode array could otherwise be a good choice for EVA + IP II malformation.

The scientific evidence is in favour of the A value measurement in modern cochlear implant therapy. For this, we need highly developed preoperative software tools such as OTOPLAN for easy assessment of cochlear size using 3-D visualization. To successfully implement the preoperative visualization outcomes, the availability of electrode arrays in different lengths for all different types of cochleae is essential.

## 5. Conclusions

Most EVA cases occur with IP II malformation. The basal turn of the cochlea may be smaller in EVA cases than in patent cochleae. Electrode selection should be adjusted accordingly. The FLEX28 seems to be good for IP II cases; however, to avoid placing the electrode in the cystic apex, a medium length electrode such as a 24mm long electrode would be a safe option.

## Figures and Tables

**Table 1:** Comparison of the mean A, H, and B values between the groups with patent cochleae, EVA only, and EVA + IP II.

	A Value—Diameter, mm	H Value—Height, mm	B Value—Width, mm
**Patent cochlea**	8.790 (7.4–9.7)	2.585 (2.0–3.2)	6.383 (5.4–7.2)
**EVA only**	8.525 (7.8–9.5)	2.675 (2.1–3.2)	6.081 (5.4–6.9)
**EVA + IP II**	8.541 (7.5–9.2)	2.651 (1.9–3.3)	6.078 (4.6–7.1)

**Table 2:** Comparison of the cochlear parameters between the left and right ear in the groups with patent cochleae, EVA only, and EVA + IP II.

		A Value—Diameter, mm	H Value—Height, mm	B Value—Width, mm
Patent cochleae	Mann–Whitney U	3,054,000	2,825,500	2,598,500
	*p*-value (2-sided)	0.924	0.367	0.089
EVA only	Mann–Whitney U	25,000	23,500	29,000
	*p*-value (2-sided)	0.461	0.370	0.750
EVA + IP II	Mann–Whitney U	597,000	680,000	622,000
	*p*-value (2-sided)	0.341	0.961	0.498

**Table 3:** Comparison of the cochlear parameters between males and females in the group with patent cochleae and EVA with IP II.

		A Value—Diameter, mm	H Value—Diameter, mm	B Value—Width, mm
Patent cochleae	Mann–Whitney U	2,891,000	2,852,000	2,670,000
	*p*-value (2-sided)	0.567	0.476	0.175
EVA + IP II	Mann–Whitney U	419,500	508,500	514,500
	*p*-value (2-sided)	0.140	0.700	0.755

## Data Availability

Data are contained within the article.

## References

[1] Patel N.D., Ascha M.S. (2018). Morphology and cochlear implantation in enlarged vestibular aqueduct. Am. J. Otolaryngol..

[2] Colvin I.B., Beale T. (2006). Long-term follow-up of hearing loss in children and young adults with enlarged vestibular aqueducts: Relationship to radiologic findings and Pendred syndrome diagnosis. Laryngoscope.

[3] Dhanasingh A., Erpenbeck D. (2021). A novel method of identifying inner ear malformation types by pattern recognition in the mid modiolar section. Sci. Rep..

[4] Khurayzi T., Almuhawas F. (2021). A novel cochlear measurement that predicts inner-ear malformation. Sci. Rep..

[5] Dhanasingh A., Jolly C. (2017). An overview of cochlear implant electrode array designs. Hear Res..

[6] Sun B., Dai P. (2015). Study on 2747 cases of inner ear malformation for its classification in patient with sensorineural hearing loss. J. Clin. Otorhinolaryngol. Head Neck Surg..

[7] Sennaroğlu L., Atay G. (2014). A new cochlear implant electrode with a "cork"-type stopper for inner ear malformations. Auris Nasus Larynx..

[8] Högerle C., Englhard A. (2022). Cochlear Implant Electrode Tip Fold-Over: Our Experience with Long and Flexible Electrode. Otol. Neurotol..

